# Raman-based spectrophenotyping of the most important cells of the immune system

**DOI:** 10.1016/j.jare.2021.12.013

**Published:** 2022-01-04

**Authors:** Aleksandra Borek-Dorosz, Anna Maria Nowakowska, Patrycja Leszczenko, Adriana Adamczyk, Anna Pieczara, Justyna Jakubowska, Agata Pastorczak, Kinga Ostrowska, Marta Ząbczyńska, Karol Sowinski, Wieslaw Ignacy Gruszecki, Malgorzata Baranska, Katarzyna Maria Marzec, Katarzyna Majzner

**Affiliations:** a Jagiellonian University, Faculty of Chemistry, Krakow, Poland; b Jagiellonian University, Jagiellonian Centre for Experimental Therapeutics (JCET), Krakow, Poland; c Medical University of Lodz, Department of Pediatrics, Oncology and Hematology, Lodz, Poland; d Maria Curie-Sklodowska University, Department of Biophysics, Institute of Physics, Lublin, Poland; e Lukasiewicz Research Network - Krakow Institute of Technology, Krakow, Poland

**Keywords:** T cells, B cells, Confocal Raman imaging, Spectroscopic markers, Carotenoids

## Abstract

**Introduction:**

Human peripheral blood mononuclear cells (PBMCs) are a heterogeneous population of cells that includes T and B lymphocytes. The total number of lymphocytes and their percentage in the blood can be a marker for the diagnosis of several human diseases. Currently, cytometric methods are widely used to distinguish subtypes of leukocytes and quantify their number. These techniques use cell immunophenotyping, which is limited by the number of fluorochrome-labeled antibodies that can be applied simultaneously.

**Objective:**

B and T lymphocytes were isolated from peripheral blood obtained from healthy human donors.

**Methods:**

The immunomagnetic negative selection was used for the enrichment of B and T cells fractions, and their purity was assessed by flow cytometry. Isolated cells were fixed with 0.5% glutaraldehyde and measured using confocal Raman imaging. K-means cluster analysis, principal component analysis and partial least squares discriminant methods were applied for the identification of spectroscopic markers to distinguish B and T cells. HPLC was the reference method for identifying carotene in T cells.

**Results:**

Reliable discrimination between T and B lymphocytes based on their spectral profile has been demonstrated using label-free Raman imaging and chemometric analysis. The presence of carotene in T lymphocytes (in addition to the previously reported in plasma) was confirmed and for the first time unequivocally identified as β-carotene. In addition, the molecular features of the lymphocytes nuclei were found to support the discriminant analysis. It has been shown that although the presence of carotenoids in T cells depends on individual donor variability, the reliable differentiation between lymphocytes is possible based on Raman spectra collected from individual cells.

**Conclusions:**

This proves the potential of Raman spectroscopy in clinical diagnostics to automatically differentiate between cells that are an important component of our immune system.

## Highlights

• Raman spectroscopy was able to differentiate between white blood cells.
• Bands of carotenoids and nucleic acids are specific Raman markers that discriminate B and T cells.
• The major carotenoid presented in T cells is β-carotene.
• The content of β-carotene depends on individual donor variability.
• Principal Component Analysis and Partial Least Square Discriminant Analysis are sufficient to discriminate T and B cells.

## Graphical abstract

**Figure:**
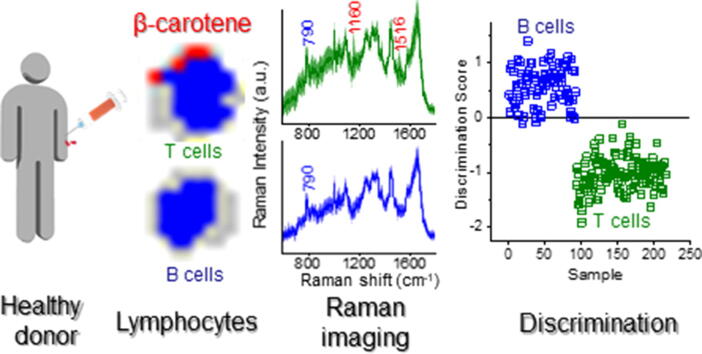

## Introduction

Lymphocytes represent the most numerous cell population within peripheral blood mononuclear cells PBMCs (70–90%) [[1], [2]], which consists of three functionally distinct cell types: T (40–70%), B (5–15%) and NK cells (5–10%). B lymphocytes differentiate into plasma cells, produce immunoglobulins, or can act as antigen-presenting cells [3]. B and T cell populations show distinct immunologic properties [[4], [5], [6]], but they are characterized by the same morphological features, including small size oscillating around 8–10 µm, a large nucleus with dense heterochromatin and cytoplasmic border containing mitochondria, ribosomes, and lysosomes [7].

The total number of lymphocytes and their percentage became diagnostic markers helpful for making an initial diagnosis of several human diseases [8]. Therefore, different methods have been widely investigated to quantify and characterize lymphocyte populations in blood samples. Eventually, in clinical practice, flow cytometry (FC) turned out to be the most powerful method of PBMCs examination and differentiation. In FC cells are classified in a process called immunophenotyping, which detects the presence or absence of a marker on the cell surface or intracellular markers specific for a particular cell type.

Despite many advantages of the multiparameter analysis of leukocyte subsets using FC the main limiting factor is the maximum number of fluorochrome-labeled antibodies, which can be used in one experiment, and the lack of possibility to measure living cells. Moreover, FC does not provide information about the intracellular localization of entities. Therefore, high-throughput and label-free techniques that could give insight into the chemical composition of cells and subcellular localization of specific biomolecules are still desirable also in clinical practice. From the beginning of the early 1990s Raman spectroscopy (RS) began to be successfully applied in the analysis of biological samples. RS allows the label-free, non-disruptive, and semi-quantitative analysis of biological samples *in situ* as well as time-lapse analysis of the molecular dynamics of a living cell [10].

Multivariate analysis of Raman spectra obtained from cytoplasm was previously used to distinguish cell subtypes within PBMCs, and the accumulation of carotenoids has been reported as an important factor that discriminates leukocytes populations [9]. Concurrently, carotenoids are a suitable biomarker for imaging since they meet conditions for signal amplification in the resonance Raman mechanism [11]. The concept of Raman spectroscopic hemogram was developed by Ramoji et al. [12], who created a classification model effectively differentiating granulocytes from lymphocytes using PCA-LDA (Principal Component-Linear Discrimination) analysis. The developed model allowed for the prediction of PBMC type with high accuracy. Subsequently, in order to reduce background originating from autofluorescence of the biological samples, Chen et al. [13] used Wavelength Modulated RS for effective separation of the CD4+ T lymphocytes and CD8+ T lymphocytes from CD56+ NK cells. In 2016 Hobro et al. [14] further improved Raman data analysis to discriminate T and B cell lines using PLS-DA. All those previous studies clearly showed that RS could be applied for the classification and analysis of subtypes of lymphocytes. Moreover, Puppels et al. [9] showed that Raman spectra from WBC cytoplasm allow distinguishing of their different subtypes [9], [12]. The presence of carotenoids has also been reported not only in blood serum, but also in human CD4+ lymphocytes [9] and in Gall bodies in the cytoplasm [33]. However, those studies were focused on the differentiation of a relatively small fraction of cells derived from one donor. Raman analysis of single spectra of leukocytes revealed endogenous tetraterpenoids, but their identification was not reliable [16], [17], [20]. Pully et al. demonstrated the presence of carotenoids in mononuclear cells using time-lapse Raman imaging [34]. Even though from the statistical point of view, the number of data points was impressive, no identification of subtypes of mononuclear cells was done.

Besides the question of the presence of carotenoids in cells could serve as a spectroscopic marker for their distinguishing, another one is whether it could be used exclusively. Due to the resonance enhancement, the identification of tetraterpenoids is facilitated but their quantification is not trivial. To date, more than 850 naturally occurring in nature carotenoids have been described, but only ∼ 20 can be detected in human blood or tissues, and ∼ 30–50 seem to have an activity of vitamin A. Among them, the most abundant are β-carotene, lutein, lycopene, α-carotene, β-cryptoxanthin, and zeaxanthin [15]. Humans do not produce carotenoids *de novo* but absorb them with nutrition [17]. Depending on the cell type, the cellular uptake of carotenoids and retinoids is facilitated by transporters such as the scavenger receptor class B type 1, the ATP-binding cassette transporter ABCA4 and the channel-like membrane protein encoded by the stimulated by retinoic acid 6 (STRA6) [18] (Supplementary Fig. S2). All T cells, natural killer cells, monocytes, and dendritic cells subsets express STRA6 transporter but at varying intensities [19]. A higher level of STRA6 expression was found in T lymphocytes in comparison to B cells [19], which may be related with different carotenoid content.

In this work, we identify a set of Raman markers (carotenoids, nucleic acids, proteins, and lipids) for reliable differentiation between two of the most important cell fractions in the immune system, B and T lymphocytes isolated from healthy donors. We demonstrated that carotenoids, with a dominant contribution of β-carotene, are present exclusively in T cells, whereas the nuclear contribution is higher for spectra of B cells. The analysis based, in total, on approx. 100, 000 collected spectra, proves its credibility and shows the potential of RS in clinical diagnosis in distinguishing between B and T cells. Finally, we developed a detailed protocol of label-free RS measurements, data collection, pre-processing, and analysis for discrimination between different fractions of lymphocytes.

## Materials and methods

### Isolation and fixation of peripheral blood mononuclear cells (PBMCs)

**Fig. 1:**
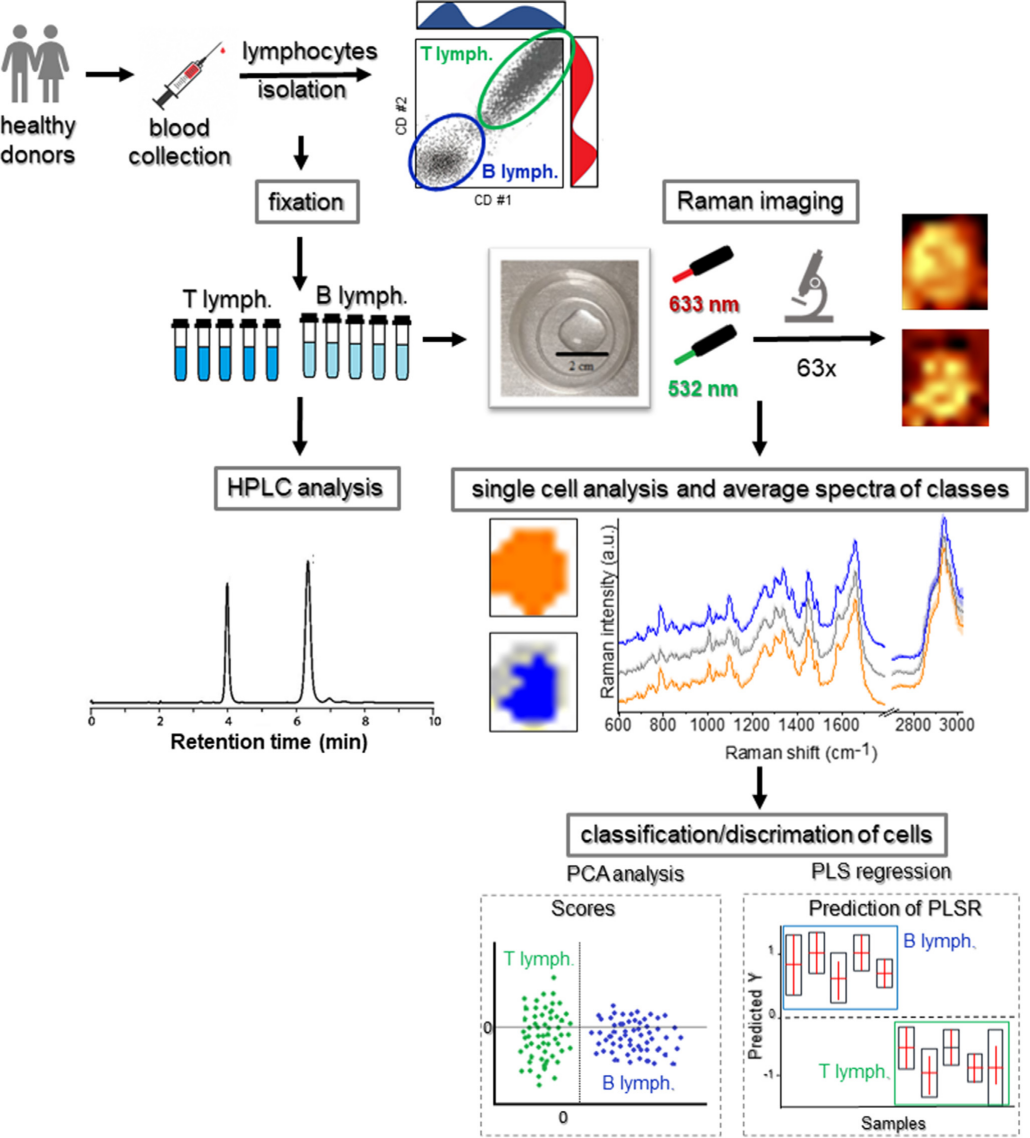
Schematic representation of a protocol for sample preparation and analysis.

B and T lymphocytes, and mononuclear cells were isolated from peripheral blood obtained from healthy donors (T and B lymphocytes, n = 5; mononuclear cells, n = 3, Fig. 1) at the Department of Pediatrics, Oncology and Hematology of the Medical University of Lodz. Samples from in total 5 healthy donors, women, age 26–41, standard diet were collected in four independent sets during 5 months. Blood from the ulnar vein was drawn on an empty stomach under the same conditions in the morning, between 7:50–8:30. Informed consent was given by each volunteer prior to the blood withdrawal and the study conformed with the principles outlined in the World Medical Association (WMA) Declaration of Helsinki, as well as in accordance with the consent of the Bioethics Committee at the Medical University of Lodz No. RNN/270/19/KE (extension KE/30/21) from 14th of May 2019.

Immunomagnetic negative selection method with EasySep™ Direct Human B Cell Isolation Kit and EasySep™ Direct Human T Cell Isolation Kit (STEMCELL Technologies Inc.) was used for the enrichment of B and T cells, respectively. This method provides untouched target cells with high viability, exceeding 90%. PBMCs were isolated by density gradient centrifugation using Histopaque®-1077 (Sigma, Deisenhofen, Germany). In the case of sample contamination with red blood cells, the erythrocytes lysis was processed by adding 1x BD Pharm Lyse™ lysing solution (BD Biosciences, San Jose, CA) and incubating 15 min at room temperature in the dark. After the washing step with PBS (without Ca^2+^ and Mg^2+^), isolated cells were fixed with 0.5% glutaraldehyde (GA) for 10 min at room temperature and then washed three times with PBS (without Ca^2+^ and Mg^2+^) to remove the excess amount of fixative. Then the cells were suspended in saline buffer and kept up at 4°C until Raman measurements for a constant period.

### Flow cytometry

**Fig. 2:**
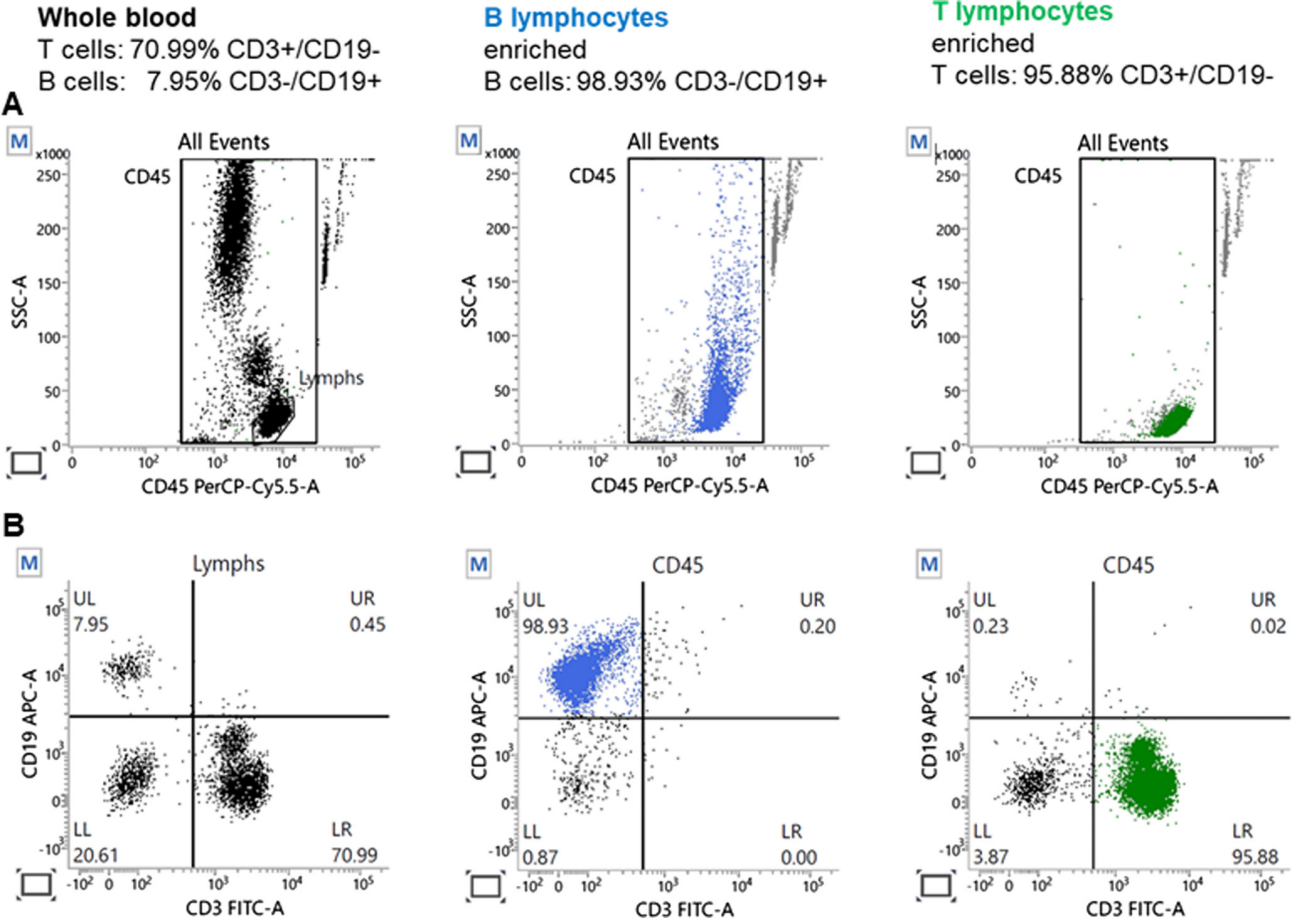
**Gating strategy for the characterization of B and T cell subsets.** The lymphocyte population (Lymphs) was identified based on side-scatter (SSC) characteristics and CD45 expression. In the whole blood B and T cells were defined further as CD19-expressing or CD3-expressing cells in the lymphocyte population (Lymphs), respectively. After magnetic separation purity of enriched lymphocytes was assessed within CD45-positive leukocytes. Cytometric analysis shows that the purity of immunomagnetic separated lymphocytes exceeded 95%. Picture presents one of the sample analysis.

The purity of isolated B cell population and T lymphocytes before RS measurements was assessed by flow cytometry. Three-colour immunophenotyping was carried out in Staining Buffer (PBS with 2% FBS) for 15 min at room temperature in the dark using the following fluorochrome-conjugated antibodies: anti-CD45- PerCP-Cy™5.5 (clone 2D1), anti-CD3-FITC (clone SK7), anti-CD19-APC (clone SJ25C1; all from BD Biosciences, San Jose, CA, USA). In peripheral blood, the CD45 antigen is expressed on the surface of all human leukocytes, i.e., lymphocytes, monocytes, and granulocytes. The CD3 antigen is present on T lymphocytes, whereas the CD19 antigen is used to discriminate human B lymphocytes. Following incubation, samples were washed with Staining Buffer and acquired on FACSLyric cytometer using BD FACSuite™ software. Up to 10,000 events were acquired per sample. The percentage of T (CD3+/CD19-) and B (CD3-/CD19+) cells in whole blood was 70.99% and 7.95%, respectively (Fig. 2). After separation, the achieved purities of the samples were high, i.e., the T lymphocytes were enriched up to 95.88%, and in the case of B lymphocytes up to 98.93% (Fig. 2).

### Confocal Raman imaging

Raman imaging of single PBMCs was performed using a confocal Raman microscope WITec Alpha 300 (Ulm, Germany) (Fig. 1) equipped with the 532 and 633 nm excitation wavelengths and a CCD detector (Andor Technology Ltd, Belfast, Northern Ireland). For single-cell measurements, a 63x water immersion objective (Zeiss W Plan-Apochromat 63x, NA = 1, Oberkochen, Germany) was used what allowed the laser to focus to a diffraction-limited spot with a diameter of 325 and 386 nm for an excitation wavelength of 532 and 633 nm, respectively. Spectra of pure carotenoids were collected with the use of 100x air objective (Olympus MPlanFL N 100x, NA = 0.9, Tokyo, Japan). The spectral resolution was equal to 3 cm^−1^. In the case of the 532 nm laser, spectral images were collected with the sampling density of 1 μm for statistical measurements, 0.5 μm for depth imaging (in *z*-axis step size was equal to 1.5 μm) and 0.162 μm for high-resolution (HR) measurements, with the acquisition time of each spectrum equal to 0.5 s. In the case of 633 nm laser, images were collected with a sampling density of 3 μm in *×* and *y* directions and an integration time of 3 s. Measurements of PBMCs were carried out by applying laser power of ∼30 mW for 532 nm excitation and ∼23 mW for 633 nm excitation (measured after the beam passes through the objective). In the case of measurements of pure carotenoids, the lower laser power was applied, i.e., ∼1 mW for 532 nm, and ∼23 mW for 633 nm. For single-cell Raman imaging, the cell suspension was centrifuged, then approximately 200 µl − 500 µl of cell suspension was placed onto the CaF_2_ slide (Crystran LTD, Poole, UK, Raman grade) and placed under the objective of the microscope. Raman measurements were performed from single cells forming a monolayer on the substrate. For each sample, only morphologically undamaged, round cells with no visible signs of abnormalities were measured. For each donor and each biological replicant, approx. 50 cells per sample were imaged using RS.

### Spectral data post-processing and analysis

The first part of the analysis was conducted using Project FIVE 5.1 Plus software (WITec GmbH, Germany). Spectral pre-processing included removal of artifacts from cosmic radiation, subtraction of background contributions, and residual autofluorescence (polynomial fitting, 3^rd^ order for the green laser, and 2^nd^ order for the red laser). Then, k-means cluster analysis (KMCA) was performed using the Manhattan distance calculation. This approach enabled spectra grouping into classes based on their similarities and extraction of the average spectra reflecting the major biochemical classes of cells, originated from the whole cell cluster or the nucleus and cytoplasm fractions (Fig. 1).

Further chemometric analysis was performed on the average spectra obtained from the whole cells or average spectra of cell nucleus or cytoplasm using Unscrambler X 10.3 software (64-bit, CAMO Software AS., Norway). Before analysis, average spectra were smoothed (Savitzky–Golay, 3rd order polynomial, 15 pts), baseline corrected and normalized (unit vector normalization), and such pre-processed spectra were subjected to multivariate Principal Component Analysis (PCA). PCA was performed to detect sources of variability in Raman spectra of investigated cells. As a result of PCA, score plots presenting the grouping of PBMCs spectra and loadings, providing information of the sources of variance in the Raman spectra of leukocytes were obtained. The final data presentation was obtained using OriginPro 2020 (OriginLab). Additionally, the ImageJ equipped with the Volume Viewer application was used for the 3D visualization of cell morphology.

Partial least square (PLS) regression, which is a supervised chemometric method, was applied in order to construct a discriminant model between B and T lymphocytes based on their spectral profile. In the first step, about 70% of the data was used to build the model and validate it. The model was constructed in a wide spectral range (700–1800 and 2800–3030 cm^-1^) with the following pre-processing of Raman spectra: constant offset elimination, multiplicative scattering correction, and internal standard. Then on 30% of the data, the model was tested. Results of PLS analysis were compared with results obtained with the use of PCA. Analysis was performed using OPUS 7.0 software (Bruker Optik GmbH, US).

### High-performance liquid chromatography

HPLC analysis of carotenoids was performed on a Nexera LC-40 (Shimadzu, Kyoto, Japan) UHPLC system equipped with a diode-array detector SPD-M40, FCV-0607H high-pressure flow-line selection valve, CTO-40C column oven, SIL-40C XR autosampler, and LC-30D XR pump. The isocratic separation was carried out on a reversed-phase Kinetex (Phenomenex Inc, Torrance, USA) core–shell column, 150 mm × 3 mm i.d., particle size 2.6 µm (Phenomenex Inc, Torrance, USA), equipped with a Security Guard C18 3 × 4 mm i.d. (Phenomenex Inc, Torrance, USA). The autosampler was set to 4°C, and the column was thermostated at 30°C. The mobile phase consisted of methanol and methyl *tert*-butyl ether (95:5, v:v). Injection volume was 5 µl, the flow rate 0.8 mL/min, and runtime 15 min. The peak spectra were scanned from 300 to 800 nm.

The isolation of carotenoids from B and T lymphocytes was carried out as follows. The cells were pelleted 1,000 × g, 4°C, 2 min. The cell pellet was homogenized and carotenoids extracted using 2 mL of ice-cold acetone. The extract was filtered using a 0.22 µm PTFE syringe filter. The acetone extract was dried under a steady stream of argon. Dried samples were redissolved in 96% ethanol and immediately injected into the HPLC system.

## Results and discussion

### Spectrophenotyping of B and T lymphocytes

**Fig. 3:**
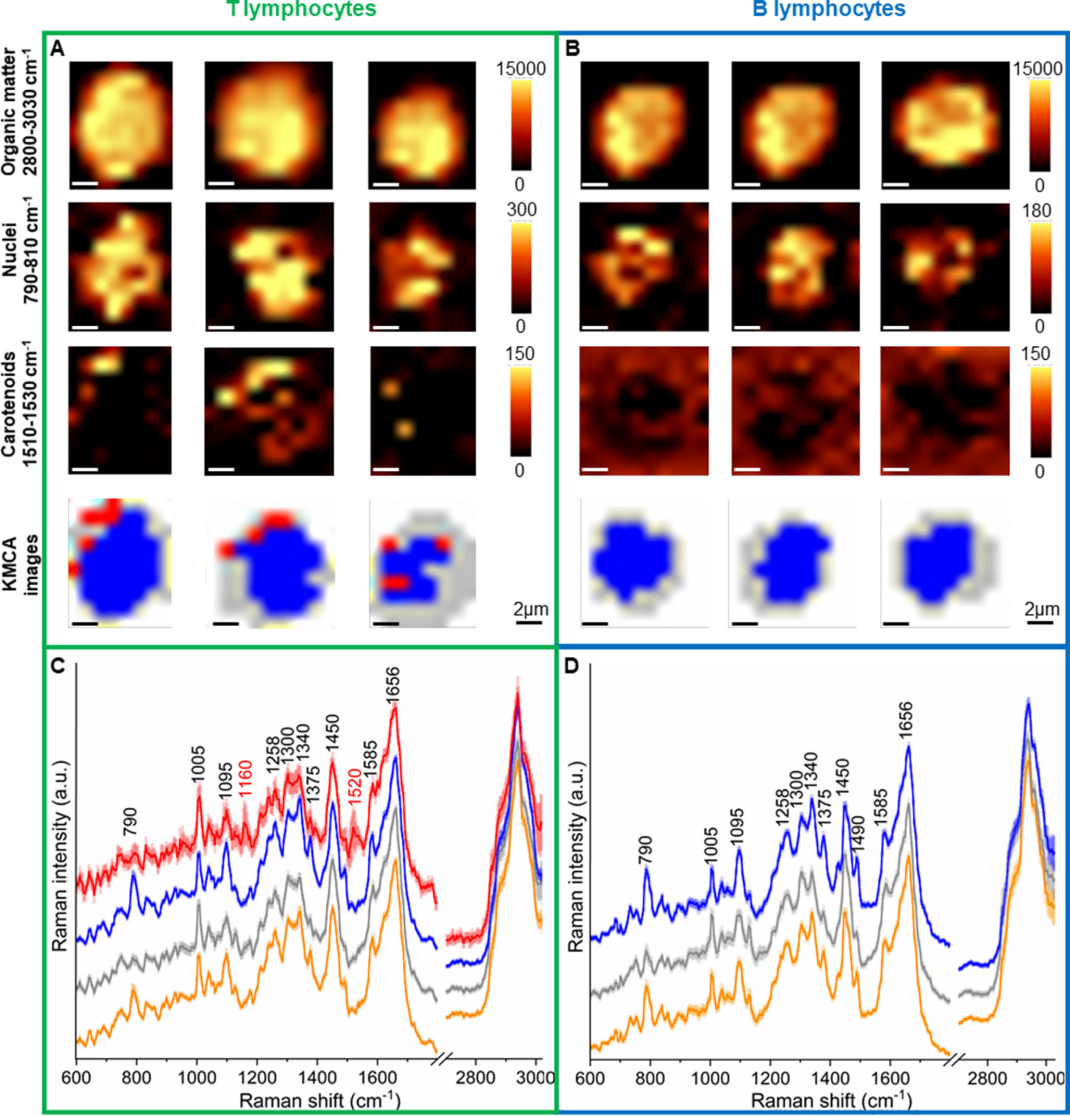
Representative chemical Raman images and corresponding false-color KMC maps for B and T cells (A-B) showing the distribution of main subcellular components: organic matter (2800–3030 cm^−1^), nucleus (790–810 cm^−1^), and carotenoids (1510–1530 cm^−1^). Average spectra with SD of all extracted classes from 213 and 501 T (C) and B lymphocytes (D), respectively. Raman images were collected from samples of 5 healthy donors using 532 nm laser. Scale bar equals to 2 µm. Raman spectra in the fingerprint region (600–1800 cm^−1^) were multiplied by 2 for better visualization. Assignment of KMC classes are as follows: the whole cell - orange, nucleus - blue, cytoplasm - grey, and carotenoids - red.

Representative Raman images of B lymphocytes (blue panel) and T cells (green panel) obtained with 532 nm excitation are presented in Fig. 3. To visualize the size and shape of cells, the Raman bands corresponding to the C-H stretching vibrations (2800–3030 cm^−1^) were integrated (Fig. 3A-B). A single Raman spectrum contains a complete information about the structure of individual subcellular components, therefore, the visualization of individual cellular organelles is possible. The nucleus was imaged by integrating the Raman band in the range of 790–810 cm^−1^, which corresponds to DNA/RNA modes (Fig. 3A-B), whereas carotenoids were visualized by integrating their marker band in the spectral range of 1510–1530 cm^−1^, related to ν_1_ mode (Fig. 3A-B). Raman maps created by the integration of individual bands were compared with the color-coded KMCA maps representing major subcellular structures of interest, i.e. nucleus (blue), carotenoids (red), and cytoplasm (grey) from which the average spectra were extracted (Fig. 3C-D). In total, randomly selected 297 B cells and 464 T cells collected from 5 different donors were spectroscopically analyzed. It translated into the analysis of approximately 76,100 spectra of B lymphocytes and T lymphocytes in total, using 532 nm excitation and 25, 366 spectra using 633 nm excitation. While carotenoids class was observed for a significant proportion of studied T lymphocytes (Fig. 3A), it was found only in a few B cells. This observation led to the hypothesis that the presence of carotenoids may be a contributing factor in distinguishing B and T cells. To take advantage of the full spectral information about the molecular structure of the studied cells (1,024 variables *per* each registered spectrum, characterizing lymphocytes in the case of 532 nm laser), multivariate chemometric analysis (PCA and PLS) was used to classify B and T lymphocytes.

**Fig. 4:**
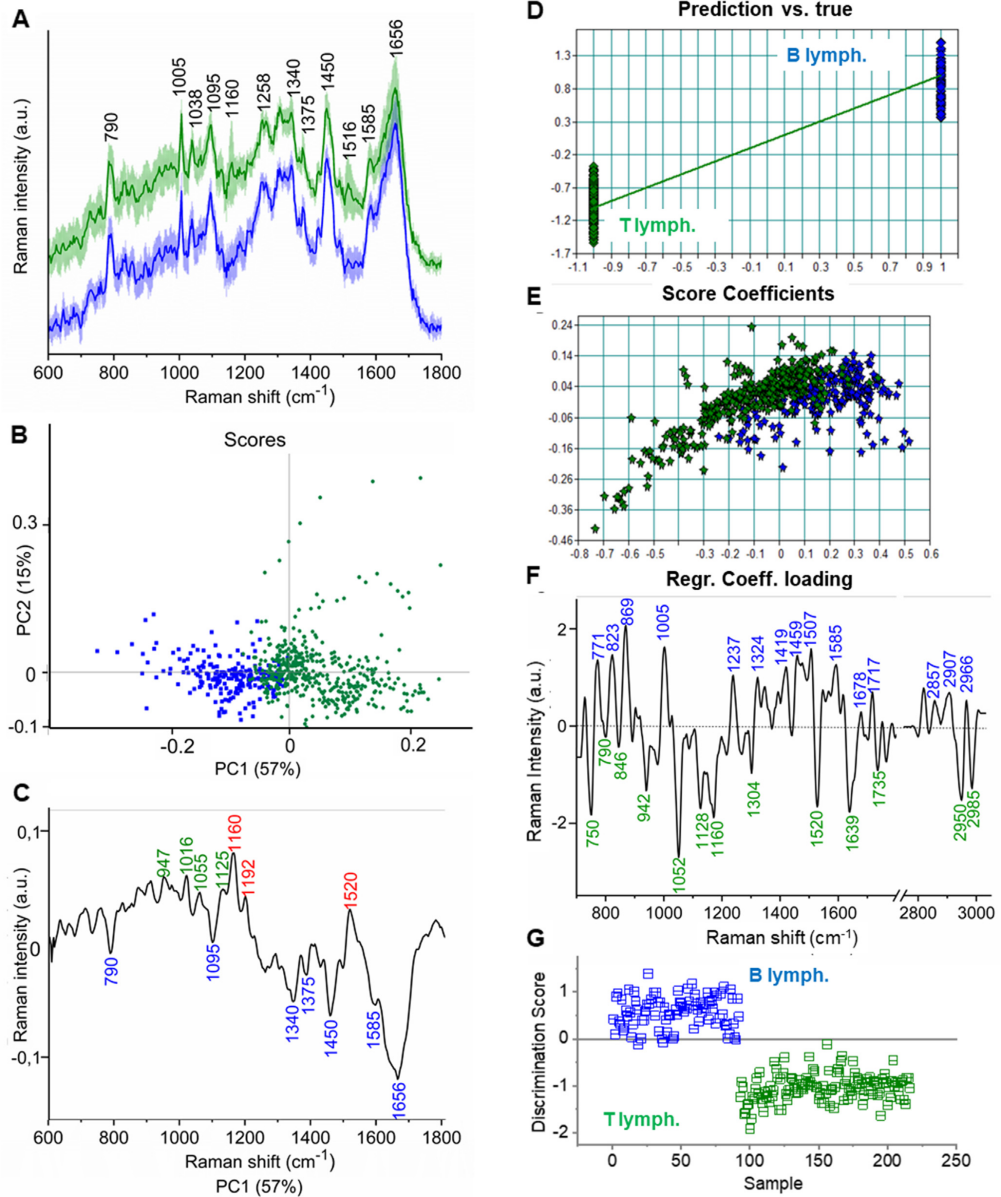
PCA (A-C) and PLS (D-G) analysis of B and T lymphocytes. The average spectra with standard deviation for whole cell cluster obtained from KMCA (A). Loadings plot (B) and scores plot (C) from PCA. PLSR model for the average spectra of B and T cells (D-E) and regression coefficient plot (F). The PCA and PLS were carried out for 297 of B cells and 464 spectra of T cells isolated from the blood of 5 donors. PCA was done for the whole-cell class, whereas PLS for the cytoplasm class only.

Both PCA (Fig. 4A-C) and PLS (Fig. 4D-G) were applied for the discrimination and classification of Raman spectra of B and T cells. First, average whole-cell and cytoplasm spectra of all B and T cells from five donors were subjected to the PCA. PCA in the fingerprint spectral range (600–1800 cm^−1^) was the most sufficient for B and T cells discrimination (Fig. 4). The analysis result consists of a PCA map (Fig. 4B) and loading plots (Fig. 4C). PLS-DA, contrary to PCA, is a supervised chemometric technique that allows optimizing separation and discrimination between different groups of samples with respect to the characterization of their molecular profiles (e.g., nucleic acids, proteins, lipids, and carotenoids). The PLS regression (PLSR) with validation of 30% of the external spectra was used to build the discrimination model (Fig. 4). A five-component PLSR model was fitted and validated by excluding random subsets of the data set. The validated model resulted in a root-mean-square error (RMSE) of 0.167 and a correct classification rate of 81.5% for B and T cells detection.

In the scatter plot of PCA analysis (Fig. 4B), the spectral separation was obtained with respect to the first component (PC-1, which explains 57% of total variance). Most spectra of B lymphocytes (blue dots) were grouped along PC-1(-), whereas T lymphocytes mostly in PC-1(+) and around 0 value at the biplot (scores), which indicates relatively low variability between spectra. A similar separation of spectra of B and T cells is shown in a score plot of PLS analysis (Fig. 4E). Even though some fraction of spectra collected from T cells mix with spectra collected from B cells, PCA and PLS discriminated these two subtypes of lymphocytes. Analysis of the loading plot of PCA provides spectral profile characterizing B and T cells. Raman features, which are positively correlated with the PC-1 loading plot, characterize T cells (green dots on scores plot), and negatively correlated bands in the PC-1 loading plot characterize B cells (blue dots on scores plot, Fig. 4B & C). Information about Raman bands characterizing B and T cells can also be obtained from regression coefficient plot of PLS (Fig. 4F).

Both PCA and PLS analyses showed that T lymphocytes can be distinguished from B cells based on the Raman bands at 1016, 1160, and 1520 cm^−1^ assigned to carotenoids (δC=CH, νC-C and νC=C, respectively), and due to increased intensities of Raman features at 947 (CH_3_ deformation), 1055 (νC–N) and 1192 cm^−1^ that originate mostly from lipids. In addition, PLS indicated bands at 982, 1304, 1435, 1735 cm^−1^, characteristic mainly for lipids and characterizing T cells. The spectra of B cells, grouped on the negative side of PC-1 axis, are characterized by Raman features originated from nucleic acids: 728 (adenine), 790 (cytosine), 1095, 1340 (CH deformation), 1375 (thymine), 1585 cm^−1^ (adenine, guanine), and proteins: 1450 (C-H bending), 1656 cm^−1^ (amide I) [34]. PLS also pointed out the bands characteristic for proteins at 869, 1005, 1237, 1324, 1459, 1678 cm^−1^, which characterize B cells.

The PCA together with PLS chemometric analyses confirmed that the presence of carotenoids, which is exclusively observed in T cells, indeed can be used as a marker to distinguish two the most important fractions of cells of the immune system. Our results show, however, that a discriminant analysis of B and T cells can also be supported by other marker bands. The nucleic acids contribution is higher for spectra of whole cell cluster for B lymphocytes in comparison to T cells (Fig. 4A). As one can see, the information obtained with the use of a supervised chemometric method, PLS, in comparison to the use of unsupervised technique, PCA, gave more complete information about the composition of B and T cells. Obtained PLS model was successfully applied on the test set of spectra to classify Raman spectra of B and T cells (Fig. 4G). The acquired correct classification rate was at the level of 88% for B and 100% for T cells detection. This demonstrates the greater suitability of supervised methods for analyzing spectroscopic data in the context of developing Raman-based algorithms for automated identification of specific cell types in blood samples.

**Fig. 5:**
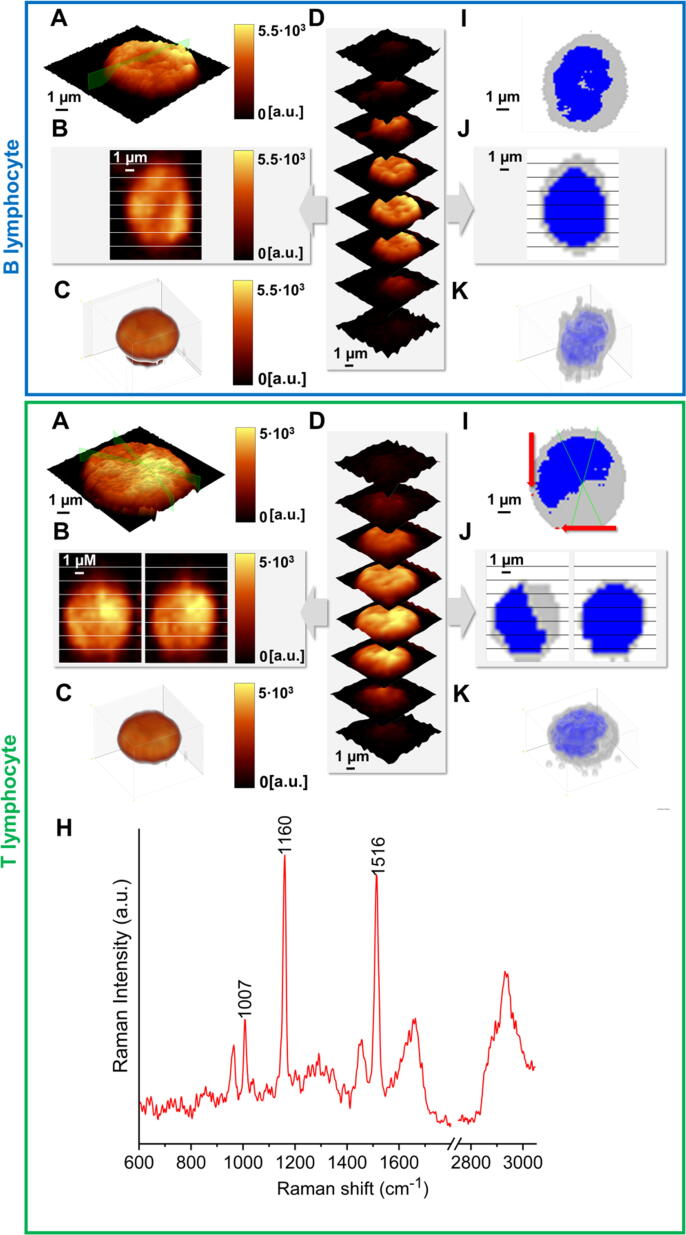
Confocal 3D Raman images of a single T (green panel) and B lymphocytes (blue panel). Raman distribution images for organic matter (integration over the bands in 2800–3030 cm^−1^ range): for high-resolution imaging (A), depth profiles *via* vertical cross-sections marked in A (B), and stack imaging obtained from layers collected every 1.5 µm step in the *z*-direction (D), 3D reconstruction of a representative cell (C). KMCA of Raman images: high-resolution imaging (E), depth profiling (F), and 3D reconstruction of a cell, which combined collected stacks of a cell (G). Raw Raman spectrum of carotenoids found in T cell is presented in H.

To confirm the differences in biochemical composition of B and T lymphocytes identified by PCA and PLS analysis, 3D imaging of T cells (Fig. 5, Supplementary Fig. S1) and B cells was done. The confocal Raman imaging enables both label-free measurements of the whole cell volume or at the selected focus plane with high accuracy. In Fig. 5 there are presented high-resolution (HR, Fig. 5A & E) and 3D (Fig. 5 B,C & D) images of T lymphocytes obtained with the 633 nm excitation. To investigate the distribution of lipids, nucleic acids, and carotenoids throughout the T cell volume, 3D imaging was performed in layer-by-layer acquisition at 1.5 μm steps in the *z*-direction from top to bottom of the cell (Fig. 5D & H). Raman 3D images were reconstructed from 2D images collected in the *xy*- plane (Fig. 5C). Analysis of Raman images obtained by integration in the spectral range characteristic for C-H stretching vibrations (2800–3030 cm^−1^ showing the spatial distribution of the organic matter, Figure 5 A-D) revealed the morphology of the whole T cell (Fig. 5C). For all confocal measurements (HR imaging and stacks), the KMCA was carried out (Fig. 5E & F, respectively) to visualize the nucleus and cytoplasm both in the *xy*- and the *xz*-plane. HR imaging of representative T cell (Fig. 5E-H) disclosed the presence of carotenoids within the cytoplasm based on their characteristic bands, i.e., 1520 cm^−1^ (stretching vibrations of C = C groups) and 1160 cm^−1^ (stretching vibrations of C-H groups). No spectral signature of carotenoids was detected in the nucleus. The spectrum of carotenoids within the cell is presented in Fig. 5H. The same methodology was applied for T and B cells with the use of 532 nm excitation (Supplementary Fig. S1).

The complex 3D approach provided insight into the composition and morphology of lymphocytes and proved that T and B cells differ in respect to the carotenoids content and morphology of the cell nucleus, which confirmed results obtained by PCA and PLS analysis. Confocal images of representative B and T cells showed that nuclei of B lymphocytes were slightly larger in comparison to T lymphocytes. Based on the KMC images, the average area of nuclei per area of a cell for B lymphocytes was approximately 0.623 ± 0.133, while for T lymphocytes 0.586 ± 0.172. A large standard deviation of the estimation may be caused by a fairly large measurement step and possibly is attributed to different shapes of nuclei [27]. Studies performed with the use of flow cytometry evidenced that the main difference in morphology of T and B cells is a slightly larger mean diameter of B lymphocytes (at the level of 5%). Nevertheless, the observed difference was smaller than the biological variability of diameters for both B and T lymphocytes of a single donor, and the ratio of nucleus to cell diameter was at the same level (∼0.9) for these two subsets of lymphocytes [28]. As reported in the literature, the ratio of nucleus to cell diameter differs depending on the method applied. In studies with the use of light optical microscopy, this ratio varied from 0.82 to 0.90 [28], [29], but in studies with the use of electron microscopy, the ratio was at the level of 0.78 [30] and 0.56 [31]. What is more, theoretical simulations of the shape of B and T cells complemented with confocal imaging revealed higher nuclear inhomogeneities and irregular shapes of the nucleus in T lymphocytes [28]. As presented (Fig. 5 and Supplementary Fig. S1), 3D confocal Raman imaging also indicates a distinct from the spherical shape of the nucleus in T lymphocytes. Therefore, a small difference in the diameter of B and T cells and inhomogeneities in the shape of the nucleus, may also be responsible for different nucleic acid packing what has an impact on the intensities of the Raman bands originating from DNA base vibrations.

### Identification of β-carotene inside T cells

**Fig. 6:**
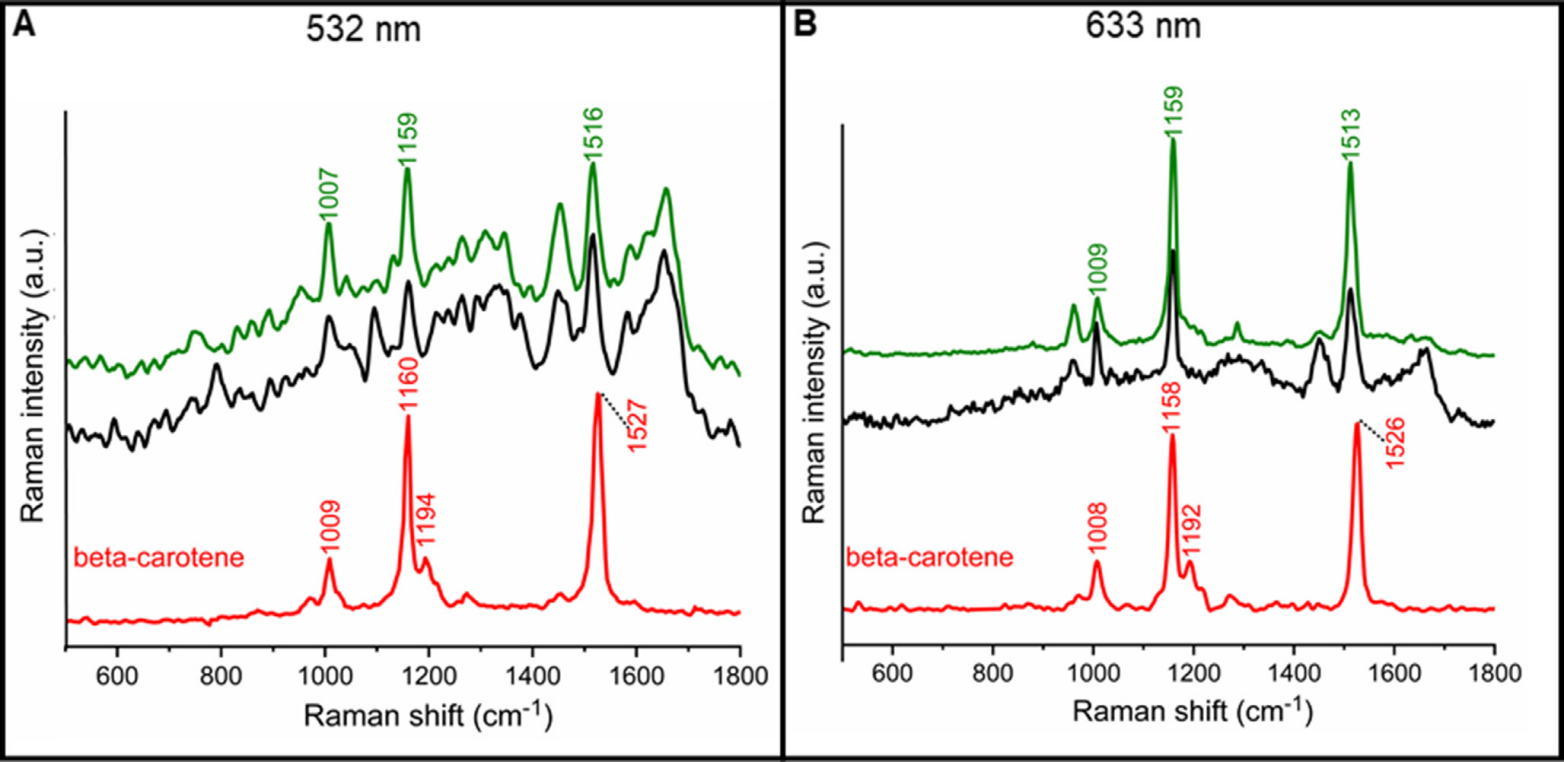
Raman spectra of carotenoids occurring in T cells (black and green) with a spectrum of β-carotene in acetone solution (red) recorded with 532 nm (left) and 633 nm (right) excitation. Spectra of cells and β-carotene in acetone solution were measured with a laser power ca. 1 mW and 23 mW, respectively. The concentration of the β-carotene solutions was: 1 mM (a) and 0.1 M (b), because with the excitation of 532 nm, the scattering was accompanied by strong absorption.

In order to identify the type of endogenous carotenoids observed in T lymphocytes (spectra marked by black and green in Fig. 6), the Raman spectra of the most abundant and expected molecules, including β-carotene (spectrum marked by red in Fig. 6), were analyzed using two excitations, i.e., 532 nm and 633 nm (Fig. 6). Based on the position of bands characteristic for tetraterpenoids in Raman spectra of T cells (1159 cm^−1^ and 1516 cm^−1^) [[22], [23]], β-carotene was indicated as a dominant carotenoid in T lymphocytes (Fig. 6). The comparison between Raman spectra collected from T lymphocytes and β-carotene solution revealed a slight shift of marker carotenoid bands (Fig. 6). That may indicate the presence of other carotenoids in the cytoplasm of T cells, besides β-carotene. However, obtained Raman spectra with 532 and 633 nm excitations are not specific enough to identify them [37].

In order to confirm that β-carotene was the most abundant carotenoid in lymphocytes T and identify endogenous carotenoids in studied PBMCs, HPLC analysis was applied on the T and B cells. Particular compounds were identified on the basis of their absorption spectra and retention time in comparison to standards. The obtained chromatogram of T lymphocytes (Supplementary Fig. S3) showed that all-trans β-carotene is the main carotenoid present in T cells. Two other carotenoids were identified in the T cells pigment extract as well: zeaxanthin and lycopene [24], [25], [26]. Several other peaks could possibly be those of carotenoids or their isomers, but were unidentifiable due to low concentrations. The chromatogram of B lymphocytes (Supplementary Fig. S3) did not show any peaks indicating the presence of carotenoids in measured samples, or their concentration was below the limit of detection. It should be noted that the applied isocratic separation conditions using a core–shell C-18 column, despite not ideal separation of all peaks, allow identification of cellular carotenoids under 7 min. It is possible that the application of core–shell columns in such studies could be beneficial both for cell and serum samples.

### Individual variability of carotenoid concentration in T cells

**Fig. 7:**
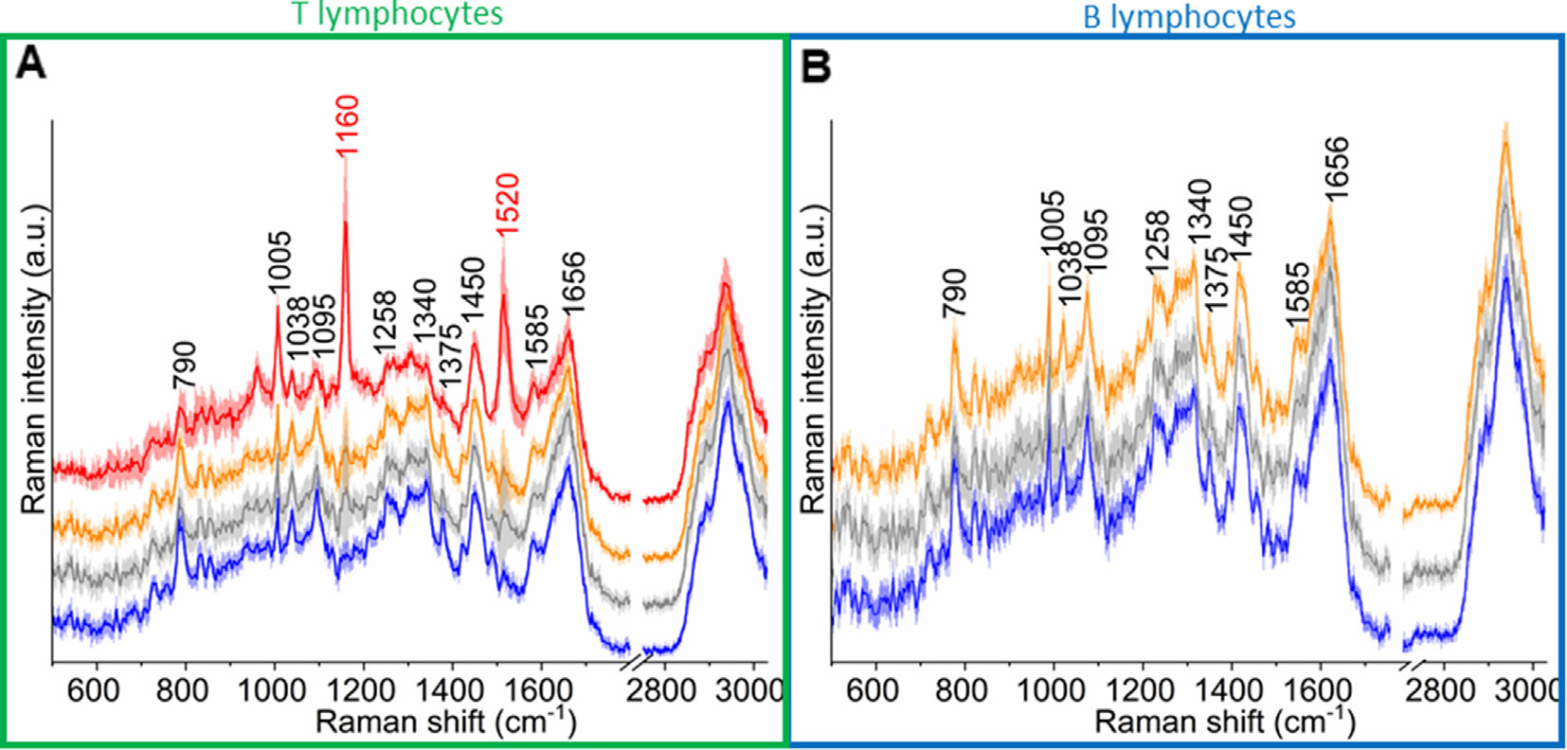
Average spectra with the standard deviation of different subcellular areas of the lymphocytes measured by using 633 nm excitation. Raman spectra of the whole cell cluster (orange), nuclear area (blue), cytoplasm (grey) and carotenoids (red) extracted from T (a) and B (b) cells. In total, 464 T cells and 297 B cells were measured, respectively. All spectra were maximally extended in the y-axis for better visualization.

The mean Raman spectra of the cell compartments acquired with the excitations of 633 with the standard deviation obtained by KMCA are shown in Fig. 7. In T lymphocytes, the marker bands of carotenoids at 1159 cm^−1^ and 1514 cm^−1^ are clearly visible (marked in red), mainly in the cytoplasm. The intensities of Raman marker bands of carotenoids were significantly higher in spectra recorded with 633 nm excitation laser line (Fig. 7) in comparison to the spectra of cells measured with the use of 532 nm laser line (Fig. 3C-D). Although the class of the carotenoids within the cytoplasm was characteristic for T cells, it can be reliably separated only in approximately 20% of measured T cells with the use of a 532 nm laser line. Carotenoids are photosensitive [20] and exhibit a resonance Raman enhancement when excited in the range of 400–550 nm [20], [22]. On the one hand, carotenoids, especially when resonantly excited in their π-π* electronic absorption transition in the visible green wavelength range, due to the resonance effect, are sensitive to radiation and show self-absorption [21]. It can result in the Raman signal decrease when the power is too high.

In order to minimize light absorption, a 633 nm excitation was applied for Raman measurements of T cells. That allowed for more efficient carotenoids detection, which was observed in approximately 30% of all T cells. However, at the same time, the signal-to-noise ratio from other biochemical components was lower than if it was a 532 nm laser. To preserve the quality of the spectra and comparable signal-to-noise ratio, the probing step size and integration time *per* spectrum were appropriately adjusted, as discussed in section Confocal Raman imaging in Results and Methods. The further pre-processing and analysis protocol were preserved for consistency of the results.

**Fig. 8:**
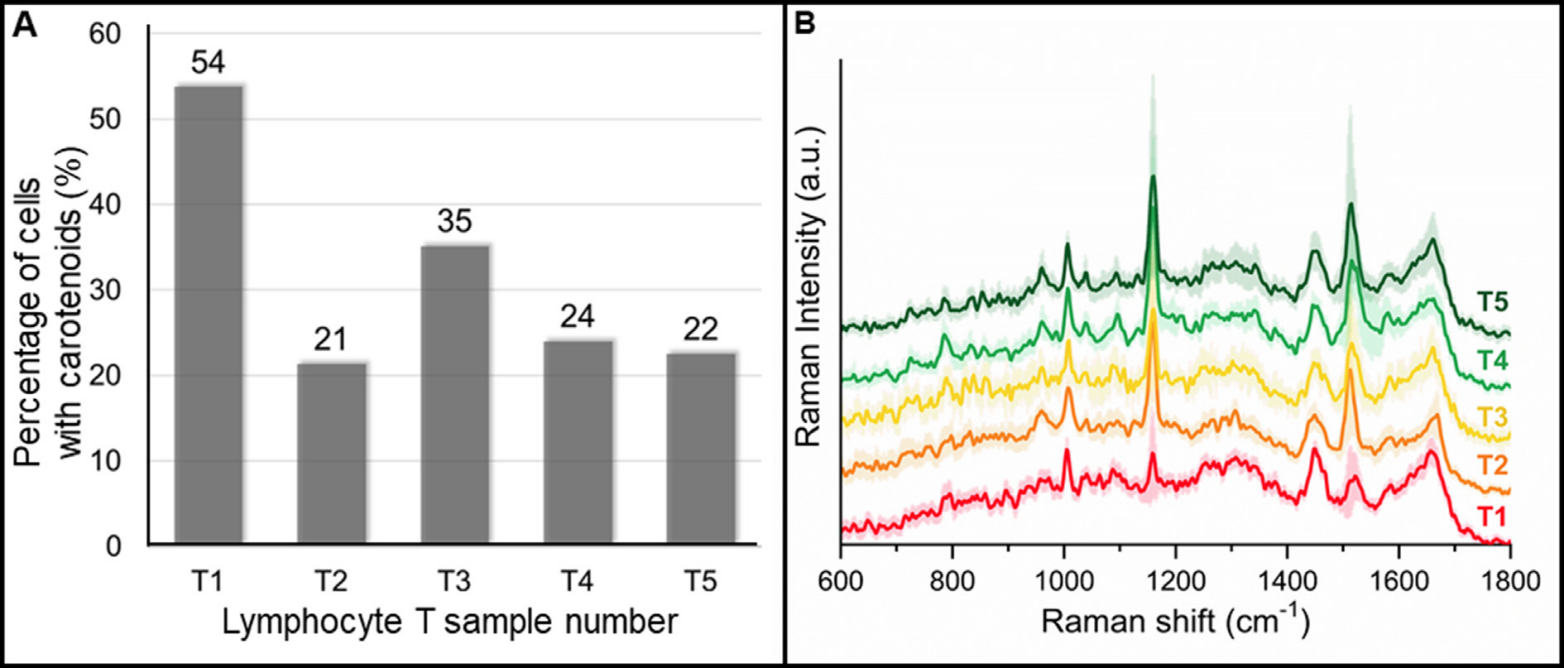
Carotenoid analysis between samples. Chart showing the percentage of cells with the presence of carotenoids Raman bands in T lymphocytes from 5 donors (A). Average spectra of the T cells cytoplasm with standard deviation for samples T1-T5 in the range of 600–1800 cm^−1^ (B).

The relatively high standard deviation of carotenoids marker bands in the spectra (Fig. 7A) indicates high variability of its concentration in T cells. In order to assess individual variability in the amount of carotenoids, the percentage of T lymphocytes in which the carotenoids were detected was calculated separately for each donor (Fig. 8A). Additionally, we compared the averaged spectra of the cytoplasm class from T lymphocytes for each donor separately to visualize and follow the variance of the carotenoid content and Raman signal intensity among all studied samples (Fig. 8B). As already mentioned, the carotenoids were observed on average in 30% of studied T cells, but the maximal number of T cells with carotenoids was defined on ca. 50% (Fig. 8A). Such an approach shows some shortcomings related to B and T cells discrimination protocol but also indicates that the presence of the carotenoids should not be a single marker for B and T cells discrimination with the application of RS. Moreover, the comparison of averaged spectra of T cells from all five donors revealed differences in the intensity of carotenoids marker bands, which in turn indicates variability in the number of carotenoids between samples. Although samples T1 and T3 showed the highest percentage of cells containing carotenoids, their intensity of marker Raman bands was low. On the other hand, in the samples T2, T4, and T5, approximately only 22% of cells exhibit carotenoids signals, but the Raman spectra of those cells manifest strong bands at 1160 and 1520 cm^−1^. Carotenoids concentration in cells not only show an individual donor variation but also, due to the resonance Raman effect, their quantification is not trivial.

T cell population is a heterogeneous group of cells, including CD4 helper cells and CD8 cytotoxic cells in various proportions. We assumed that overrepresentation of the specific T cell subpopulation could contribute to high carotenoid content observed in the sample and that tetraterpenoids concentration could be associated with different CD4 to CD8 ratios. Therefore, we assessed the detailed immunophenotypic profile of T cells and determined the percentage of the subpopulations within analyzed T cell samples. However, we did not observe any relations between the intracellular content of carotenoids and CD4 to CD8 ratio (Supplementary Information, Tab. S1). Our analysis then suggested that there is no selective carotenoids uptake mechanism that predominates in one T cell subpopulation. Conversely, the presence of higher carotenoids content is a common feature of T lymphocytes.

## Conclusions

Quantification, analysis and recognition of biochemical features of T and B lymphocytes isolated from human blood were possible using label-free Raman spectroscopy imaging. It proves that this methodical approach can support the initial diagnosis or tracking the clinical course of several human diseases. We have developed a detailed protocol for measurement, data collection, pre-processing, and analysis, which allow for differentiation of PBMCs populations obtained from a statistically significant number of healthy donors. Reliable discrimination of cells is possible based on spectral markers of carotenoids, nucleic acids, proteins, and lipids, detected at the sub-cellular level. The accumulation of carotenoids exclusively in T lymphocytes was clearly evidenced in the Raman spectra and supported by quantitative analysis carried out with the HPLC method. HPLC results indicated that β-carotene is the most abundant carotenoid in T cells. Moreover, for the first time, we have presented that although the presence of carotenoids in T lymphocytes depends on the individual donor variability, the reliable distinction between PBMCs is still possible.

Despite the fact that Raman spectrophenotyping of leukocytes could be performed by 532 nm or 633 nm laser excitation, the most effective spectral discrimination of cells was obtained using 633 nm excitation, followed by fingerprint analysis of the average spectra of individual cells by PCA and PLS methods. Presented results prove the potential of Raman spectroscopy in clinical diagnosis to automatically distinguish between B and T cells.

**Declarations:**

## Funding

This work was supported by “Label-free and rapid optical imaging, detection and sorting of leukemia cells” project, which is carried out within the Team-Net programme (POIR.04.04.00-00-16ED/18-00) of the Foundation for Polish Science co-financed by the European Union under the European Regional Development Fund. This research was partially supported by the Priority Research Area Digiworld under the program Excellence Initiative – Research University at the Jagiellonian University in Kraków and research mini-grant for young and doctoral students of the Faculty of Chemistry as part of the SciMat project, “Development of spectroscopic analytical methodology for leukocyte phenotyping’, PSP N20/MNS/000023. Authors would like to address special gratitude to Ms. Adrianna Wisłocka-Orłowska, Ms. Klaudia Mielnik and Dr. Krzysztof Brzozowski (Jagiellonian University, Faculty of Chemistry, Krakow, Poland) for their kind assistance in data acquisition.

## Ethics approval

All procedures performed in studies involving human participants were in accordance with the ethical standards in the World Medical Association (WMA) Declaration of Helsinki, as well as in accordance with the consent of the Bioethics Committee at the Medical University of Lodz No. RNN/270/19/KE (extension KE/30/21) from 14th of May 2019.

## Compliance with Ethics Requirements

All procedures followed were in accordance with the ethical standards of the responsible committee on human experimentation (institutional and national) and with the Helsinki Declaration of 1975, as revised in 2008 (5). Informed consent was obtained from all patients for being included in the study.

## Consent to participate (include appropriate statements)

Informed consent was obtained from all individual participants included in the study.

## CRediT authorship contribution statement

**Aleksandra Borek-Dorosz:** Investigation, Formal analysis, Visualization, Writing – original draft. **Anna Maria Nowakowska:** Investigation, Formal analysis, Visualization, Writing – original draft. **Patrycja Leszczenko:** Investigation, Formal analysis, Visualization, Methodology, Writing – review & editing. **Adriana Adamczyk:** . **Anna Pieczara:** Investigation, Formal analysis, Visualization, Methodology, Writing – review & editing. **Justyna Jakubowska:** Investigation, Formal analysis, Visualization, Methodology, Writing – review & editing. **Agata Pastorczak:** Investigation, Formal analysis, Visualization, Methodology, Writing – review & editing. **Kinga Ostrowska:** Investigation, Formal analysis, Visualization, Methodology, Writing – review & editing. **Marta Ząbczyńska:** Investigation, Formal analysis, Visualization, Methodology, Writing – review & editing. **Karol Sowinski:** Investigation, Formal analysis, Visualization, Methodology, Writing – review & editing. **Wieslaw Ignacy Gruszecki:** Supervision, Validation, Resources, Writing – review & editing. **Malgorzata Baranska:** Supervision, Funding acquisition, Resources, Validation, Writing – review & editing. **Katarzyna Maria Marzec:** Conceptualization, Methodology, Validation, Supervision, Writing – review & editing. **Katarzyna Majzner:** Conceptualization, Methodology, Validation, Supervision, Writing – review & editing.

## Declaration of Competing Interest

The authors declare that they have no known competing financial interests or personal relationships that could have appeared to influence the work reported in this paper.
