# Supplementary material for: Raman-based spectrophenotyping of the most important cells of the immune system
Source: J Adv Res. 2022 Jan 4;41:191–203. doi: 10.1016/j.jare.2021.12.013 (PMC9637483; doi:10.1016/j.jare.2021.12.013)
Supplement: Supplementary data 1 [file mmc1.docx]

**Raman-based spectrophenotyping of the most important cells of the immune system**

Aleksandra Borek-Dorosz^a,b^, Anna Maria Nowakowska^a^, Patrycja Leszczenko^a^, Adriana Adamczyk^a^, Anna Pieczara^b^, Justyna Jakubowska^c^, Agata Pastorczak^c^, Kinga Ostrowska^c^, Marta Ząbczyńska^c^, Karol Sowinski^d^, Wieslaw I. Gruszecki^d^, Malgorzata Baranska^a,b^, Katarzyna Maria Marzec^e^*, Katarzyna Majzner^a,b^*

^a^ Jagiellonian University, Faculty of Chemistry, Krakow, Poland

^b^ Jagiellonian University, Jagiellonian Centre for Experimental Therapeutics (JCET), Krakow, Poland

^c^ Medical University of Lodz, Department of Pediatric, Oncology and Hematology, Lodz, Poland

^d^ Maria Curie-Sklodowska University, Department of Biophysics, Institute of Physics, Lublin, Poland

^e^ Lukasiewicz Research Network - Krakow Institute of Technology, Krakow, Poland

* correspondence to:
K. M. Marzec (katarzyna.marzec@kit.lukasiewicz.gov.pl)

K. Majzner (katarzyna.b.majzner@uj.edu.pl)


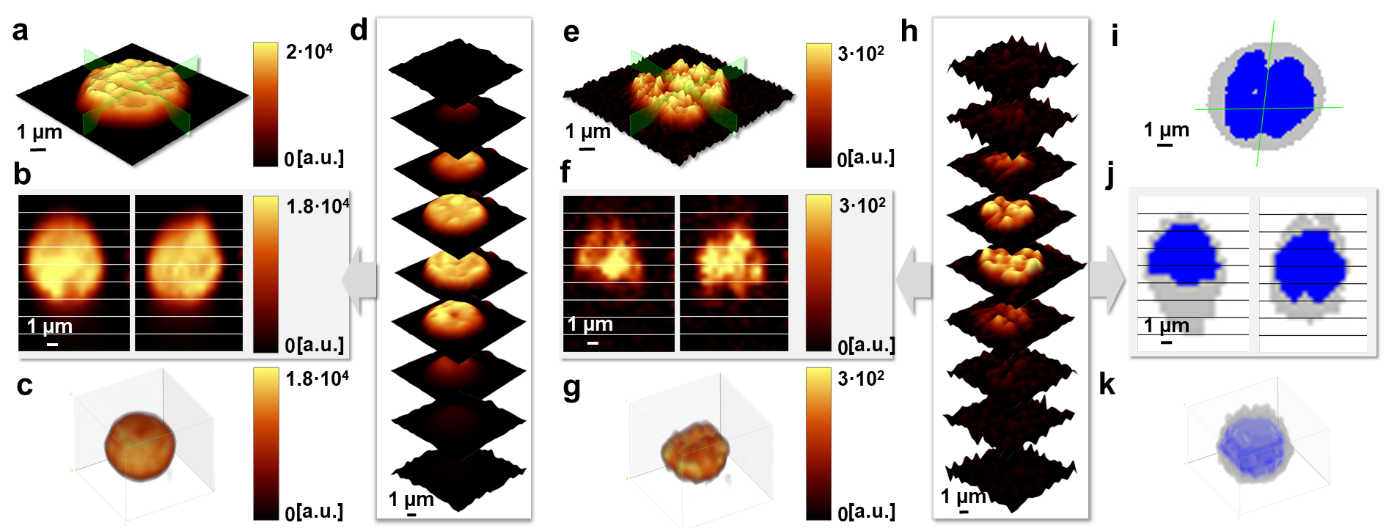


**Fig. S1. 3D Raman measurements of T lymphocyte with 532 nm excitation wavelength**. Raman distribution images for organic part (integration over bands in 2800-3030 cm^-1^ range): after high-resolution imaging (a), depth profiling (b) and stack imaging (d), 3D visualization of a whole cell (c). Raman distribution images for the nucleus (integration over the band in 780-800 cm^-1^ range): after high-resolution imaging (e), depth profiling (f) and Stack imaging (h), 3D visualization of the nucleus (g). KMC analysis of measured cell after high-resolution imaging (i), depth profiling (j) and 3D visualization of a cell after cluster analysis (k).

**Table 1. Detailed immunophenotype analysis of T cell subpopulations with respect to carotenoid content.**

| **Sample** | **Percentage of cells with carotenoids [%]** | **CD4 %** | **CD8 %** | **CD19 %** |
| --- | --- | --- | --- | --- |
| T1 | 53.7 | 43.02 | 21.85 | 5.85 |
| T2 | 21.3 | 44.68 | 38.28 | 10.85 |
| T3 | 35.1 | - | - | - |
| T4 | 24.0 | - | - | - |
| T5 | 22.5 | 43.44 | 20.96 | 7.76 |


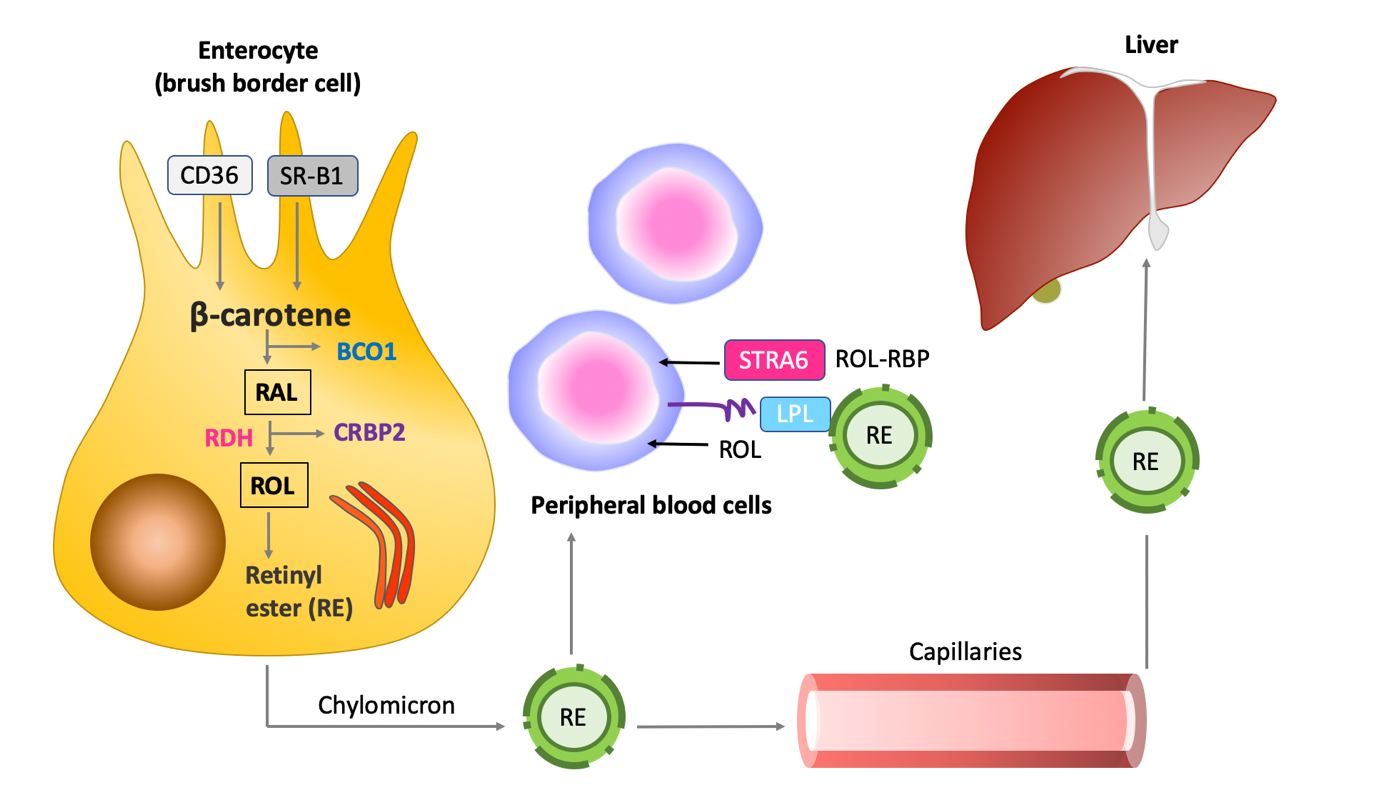


**Fig. S2. Systemic transport of carotenoids throughout the body starts from the small intestinal.** Carotenoid absorption is supported by scavenger receptor class B type 1 (SR-B1) and a cluster of differentiation 36 (CD36). β-carotene can be converted to retinal due to β-carotene-15,15′-dioxygenase (BCO1). Next, due to retinal dehydrogenases (RDH) activity, RAL undergoes transformation to retinol (ROL) which involves cellular retinol-binding protein (CRBP2). Finally, ROL is converted to retinyl esters (RE). Structures called chylomicrons are responsible for the transport of RE throughout the body. RE packaged within chylomicrons can be transported and stored in the liver or can be delivered to peripheral cells and uptake within a process mediated by lipoprotein lipase. The process of retinol uptake by lymphocytes from retinol-binding protein is mediated by STRA6 transporter.





**Fig. S3. Chromatogram from HPLC analysis of T lymphocytes.** Representative chromatogram from normal T cells (black trace) and T-cells enriched with β-carotene (green). The chromatogram of the standard of β-carotene is presented as a red curve.


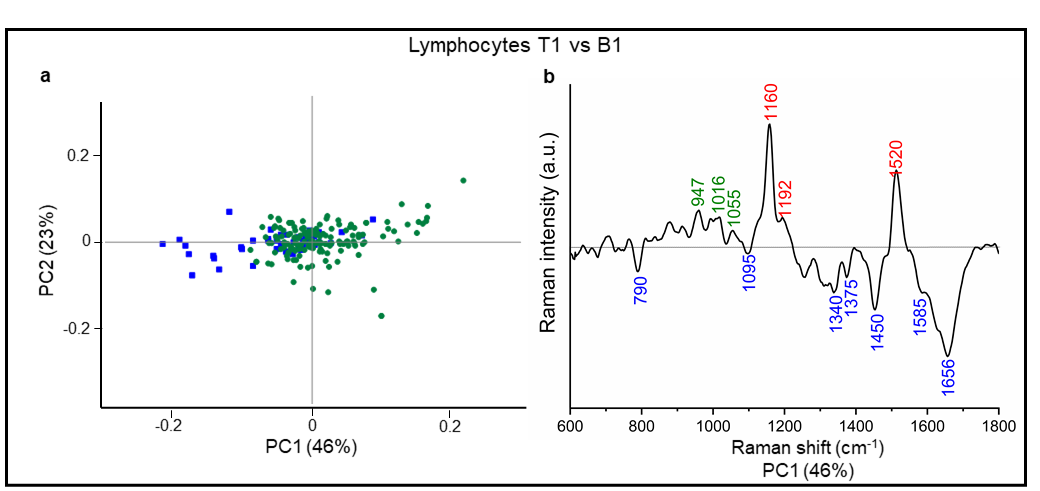

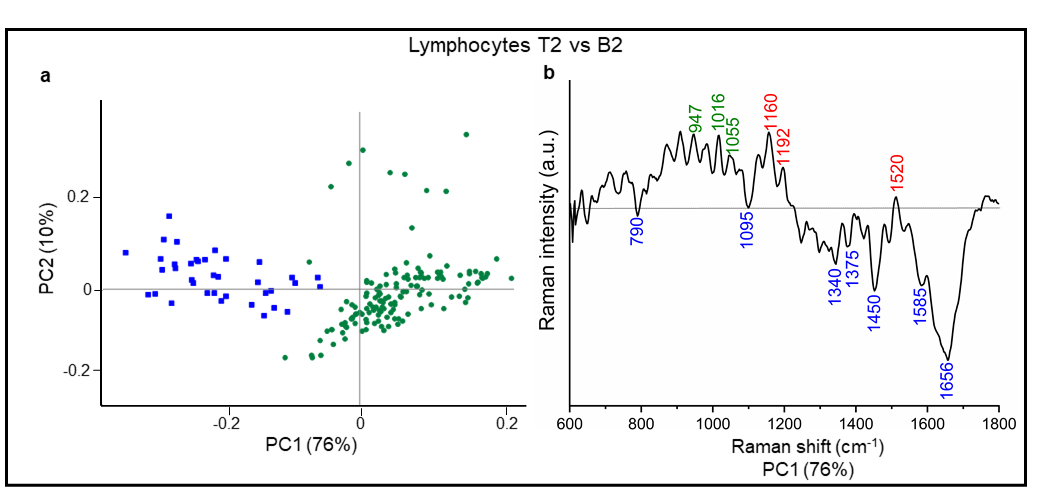

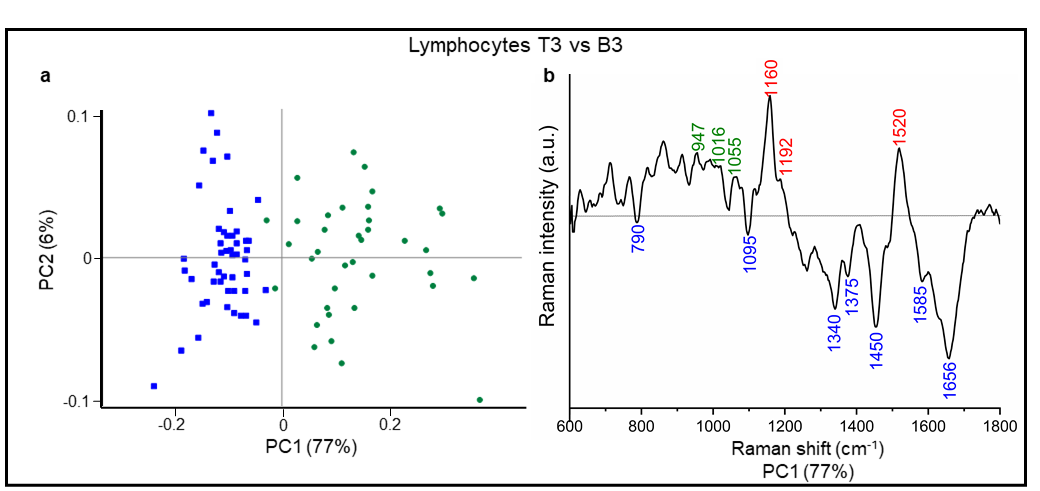

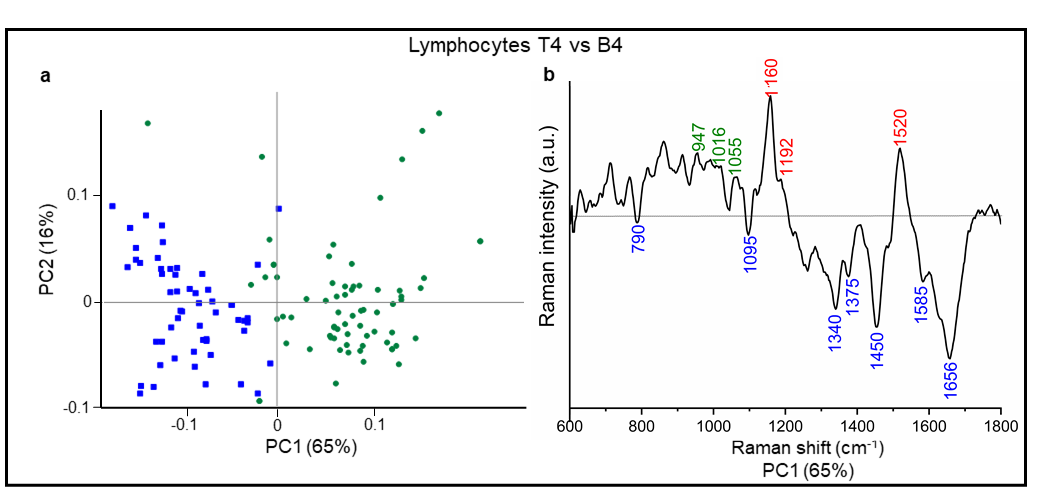

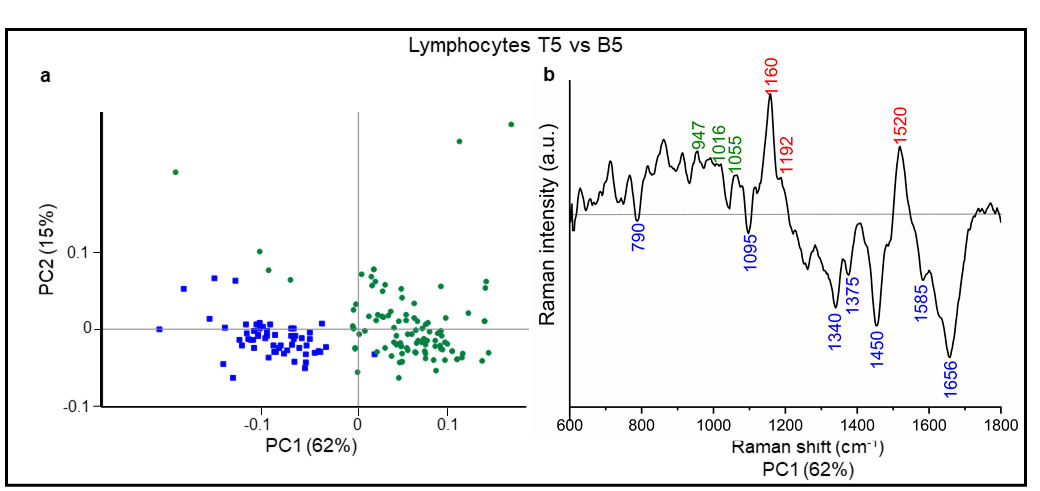


**Fig. S4. Principal component analysis of B and T lymphocytes presented separately for each donor (T1-T5).** Loadings plot (a) and scores plot (b) from PCA.
